# Practical Guide for Quantification of In Vivo Degradation Rates for Therapeutic Proteins with Single-Cell Resolution Using Fluorescence Ratio Imaging

**DOI:** 10.3390/pharmaceutics12020132

**Published:** 2020-02-05

**Authors:** Ian Nessler, Cornelius Cilliers, Greg M. Thurber

**Affiliations:** 1 Department of Chemical Engineering, University of Michigan, Ann Arbor, MI 48109, USA; inessler@umich.edu (I.N.); ccillier@umich.edu (C.C.); 2 Department of Biomedical Engineering, University of Michigan, Ann Arbor, MI 48109, USA

**Keywords:** antibody–drug conjugates, pharmacokinetic imaging, single cell measurements, residualization

## Abstract

Many tools for studying the pharmacokinetics of biologics lack single-cell resolution to quantify the heterogeneous tissue distribution and subsequent therapeutic degradation in vivo. This protocol describes a dual-labeling technique using two near-infrared dyes with widely differing residualization rates to efficiently quantify in vivo therapeutic protein distribution and degradation rates at the single cell level (number of proteins/cell) via ex vivo flow cytometry and histology. Examples are shown for four biologics with varying rates of receptor internalization and degradation and a secondary dye pair for use in systems with lower receptor expression. Organ biodistribution, tissue-level confocal microscopy, and cellular-level flow cytometry were used to image the multi-scale distribution of these agents in tumor xenograft mouse models. The single-cell measurements reveal highly heterogeneous delivery, and degradation results show the delay between peak tumor uptake and maximum protein degradation. This approach has broad applicability in tracking the tissue and cellular distribution of protein therapeutics for drug development and dose determination.

## 1. Introduction

Therapeutic proteins remain one of the fastest growing areas of pharmaceutical development in the treatment of many diseases including cancer and autoimmune disorders [1,2,3]. The varying physicochemical properties of next generation proteins, including molecular weight, molecular radius, avidity, charge, etc., can result in unexpected pharmacokinetics, making it difficult to predict their distribution [4] and subsequent efficacy. Although these agents act at the molecular scale, it is necessary to quantify both the microscopic (sub-cellular and cellular) and macroscopic (tissue and organ) distribution in order to bridge the relationship between pharmacokinetics (PK) and pharmacodynamics (PD) [5,6,7]. For example, in the case of antibody–drug conjugates (ADCs), efficacy can be enhanced by understanding the internalization/degradation and payload release at the subcellular scale, the average number and variability of payload molecules required to achieve cell death in vivo at the cellular scale [8], the number of cells in the tumor receiving a therapeutic dose at the tissue scale, and the healthy tissue exposure and resulting toxicity at the whole organ level [9]. The distribution of biologics across multiple length scales is readily measured using near-infrared (NIR) labeling to track biologics; however, to quantify protein degradation, a second NIR label is needed.

One approach uses dual NIR labeling of proteins with a non-residualizing and residualizing fluorophore. The residualization properties of many molecular labels including radiolabels and other visible light fluorophores are currently known [3,10,11]. For example, in nuclear imaging ^125^I is a non-residualizing label, which results in washout of the signal once the iodine is released, typically following protein degradation. Therefore, the signal approximates intact protein. In contrast ^111^In, ^68^Ga, ^98^Zr are residualizing agents, which approximates the cumulative uptake of the protein [12]. In an elegant approach, Ferl et al. dually labeled different engineered protein variants with residualizing ^111^In and non-residualizing ^125^I radioisotopes to measure in vivo protein degradation [13]. To model the degradation within each organ, a known quantity of each isotope was injected and the relative amount of ^125^I to ^111^In was measured over time. This method provides robust results for tracking organ biodistribution and degradation at the whole animal and organ level (e.g., [14]). Motivated by this approach, we measured the residualization properties of NIR fluorophores, identifying both residualizing and non-residualizing dyes [15]. Using a dual non-residualizing and residualizing label, the local intact and degraded protein can be detected [11,12,13,15,16]. The current protocol is similar in concept to the radiolabeling approach but uses NIR fluorescence to increase spatial and temporal resolution. This allows measurement of degradation and distribution across multiple length scales using the high spatial resolution of fluorescence and ability to quantify kinetic rates, such as degradation at the cellular level in vivo while reducing safety concerns, time/half-life constraints, and expense of radioactivity. Importantly, multiple length scales can be analyzed in vivo for the *same* animal, providing insight into heterogeneity and inter-animal variability.

Previously, we have demonstrated this technique by measuring the distribution and degradation of the FDA approved ADC Kadcyla (T-DM1) at several length scales, in vitro and in vivo [8]. We found that the distribution of a clinical dose (3.6 mg/kg) of T-DM1 in HER2 expressing tumor xenografts was highly heterogeneous and perivascular. We measured the amount of degraded ADC and corresponding release of payload and demonstrated that the targeted perivascular cells received more small molecule payloads than necessary to achieve cell death, resulting in “overkill” of the perivascular cells [8]. Additionally, we showed that efficacy and survival is improved when the same payload dose is distributed more homogeneously throughout the tumor; targeting more cells with a lower payload dose [8]. Building on the previous results, we apply the protocol to three other well-characterized proteins, epidermal growth factor (EGF), cetuximab, and anti-A33 antibody, both in vitro and in vivo to demonstrate the wide applicability of the technique for the measurement of cellular degradation and tissue distribution of other novel protein therapeutics. The method is based on the different residualization properties of two NIR fluorescent dyes, which are used to distinguish intact versus degraded protein. NIR wavelengths have low tissue autofluorescence and high tissue penetration, reducing optical artifacts [17,18]. NIR fluorescence combines the whole animal and biodistribution capabilities of radiolabels [19,20] with the tissue and cellular kinetic measurements of fluorescence [21]. We also provide an alternative dye pair (utilizing a visible light dye) to measure degradation with greater sensitivity for lower expressing targets. The ability to track the delivery of therapeutic proteins from whole animal to subcellular resolution enables investigation of the multi-scale distribution of lead compounds in vitro and in vivo and facilitates the development of predictive models for lead compound selection.

## 2. Materials and Methods

### 2.1. Cell Culture and Animals

A431, NCI-N87, and LS174T cells were cultured 2–3 times per week up to a maximum passage number of 50 and grown in RPMI 1640 or DMEM supplemented with 10% (*v*/*v*) FBS, 50 U/mL penicillin, and 50 µg/mL streptomycin at 37 °C with 5% CO_2_ based on ATCC recommendations. Annual use of the Mycoalert Testing Kit (Thermo Fisher Scientific, NC9719283) confirmed the absence of mycoplasma. All animal studies for this project (PRO00008778, approved 20/12/2018) were conducted in compliance with the Institutional Animal Care and Use Committee (IACUC) of the University of Michigan and Association for Assessment and Accreditation of Laboratory Animal Care International (AAALAC). Pharmacokinetic and in vivo tumor distribution animal studies were conducted in 4–6 week old homozygous female nude (RRID: 2175030, Foxn1nu/nu, Jackson Laboratories) mice. For in vivo tumor distribution and growth studies, the nude mice were inoculated in the flank with 1 × 10^6^ cells in Matrigel (Fisher Scientific, CB40234A).

### 2.2. Plasma Clearance

The impact of dye conjugation on the observed pharmacokinetics was measured via blood sampling. At each timepoint, 10 µL of blood was collected via retro-orbital blood sampling and added to 15 µL of PBS-EDTA (10 mM). The mixture was centrifuged at 3000× *g* for 1 min and then 18 µL of supernatant (Plasma) was collected and stored at −80 °C until all samples were collected. After completion of blood sampling, samples were thawed and scanned on the Odyssey CLx scanner or Biotek plate reader. Fluorescent values were normalized to the 1 min time point for individual mice to determine the relative impact of dye conjugation on plasma clearance.

### 2.3. Protein Fluorophore Conjugation

All proteins were conjugated via NHS ester reaction chemistry. Proteins (>2 mg/mL) were buffered with 10% (*v*/*v*) sodium bicarbonate (7.5% in PBS) to slightly increase pH of reaction for optimal labeling. Dyes were added in molar ratios approximately 1.5–2× the desired degree of labeling (e.g., 0.5 molar ratio to achieve 0.3 DoL). Reactions reached completion at 4 h and were then purified using P-6 Biogel. Briefly, 800 µL of P-6 Biogel was added to a Costar Spin-X column and centrifuged at 3500× *g* for 1 min. The filtrate (PBS) was removed and 100 µL of reaction mixture was added to the top of the column. The column was centrifuged for 1 min at 3500× *g* and the purified protein fluorophore conjugate was collected. The protein was then confirmed to be pure after running the purified protein on an SDS-PAGE gel and scanning the gel on the Odyssey CLx.

### 2.4. Degradation Assay In Vitro 

Cells were stripped from culturing flasks and plated in 96 well plates (for flow cytometry) and in 8 well chamber slides (for microscopy) at ~90% confluency then allowed to adhere to the plate overnight. During the experiment, media was replaced daily to reduce buildup of fluorescent byproducts. At each timepoint 40 nM dual labeled protein solution at a volume of 100 µL for 96 well plate or 300 µL for chamber slides was incubated for 30 min at 37 °C. The incubated wells were then aspirated and washed 2× with complete media and then the media was replaced. After the final timepoint, all cells were washed 1× with complete media and then 1× with PBS to remove all fluorophore that had leaked out of the cell. The chamber slides were then immediately imaged on a confocal microscope while each well of the 96 well plate was incubated in 100 µL of 0.05% Trypsin-EDTA until cells were detached (~10 min). Cells were gathered from each well and subsequently washed 2× with PBS/BSA before resuspending in PBS, passing through a 40 µm filter to remove cell clumps, and running on the Attune flow cytometer. To convert fluorescence signal to number of antibodies per cell, Quantum Simply Cellular anti-human beads (Bangs Laboratories, Fishers, IN) were labeled according to the manufacturer’s instructions using the dually labeled antibodies. 

### 2.5. Degradation Assay Ex Vivo

Once tumor xenografts reached a volume of ~300–500 mm^3^, 3.6 mg/kg of dual labeled T-DM1 was injected via tail vein. Mice were sacrificed at 24, 48, or 72 h after injection and the tumor was resected. The tumor was cut into small pieces and then incubated in 5 mL of 5 mg/mL collagenase IV-PBS solution for 25 min at 37 °C. After incubation, 5 mL of RPMI 1640 complete media was added to the cell suspension, the cells were pelleted at 300× *g* for 5 min and the supernatant was removed. The cell pellet was resuspended in PBS and passed through a 40 µm filter to remove clumped cells then analyzed on the Attune flow cytometer.

## 3. Results

**Figure 1:**
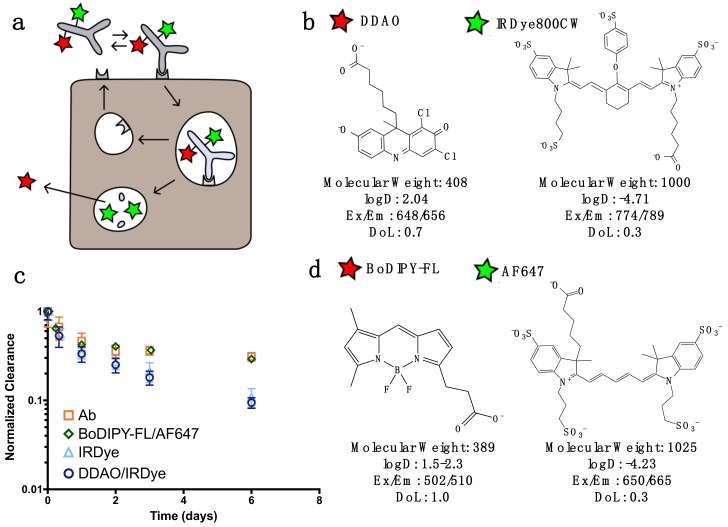
Dual label near-infrared (NIR) fluorescence imaging technique concept. (**a**) Graphic depiction of dually labeled antibody binding the cell, internalizing, and degrading. The non-residualizing DDAO (red star) leaks out of the cell following protein degradation, while the residualizing IRDye (green star) is trapped. (**b**) DDAO and IRDye dye chemical structures, molecular weights, maximum excitation/emission, and logD (pH 7.4) calculated by MarvinSketch. (**c**) The plasma concentration over time of trastuzumab-IRDye, dually labeled (DDAO/IRDye or BoDIPY-FL/AF647) trastuzumab and trastuzumab (Ab, measured by ELISA). Trastuzumab DDAO/IRDye is cleared at the same rate when compared to trastuzumab-IRDye800 (which is equal to unlabeled trastuzumab over 3–4 days) [23]. The impact of BODIPY-Fl and AF647 is negligible over 6 days. (**d**) Structure and optical properties for AF647 and BoDIPY-FL with BoDIPY-FL. The logD values for BODIPY-Fl are taken from the literature [27]. DoL, degree of labeling.

**Figure 2:**
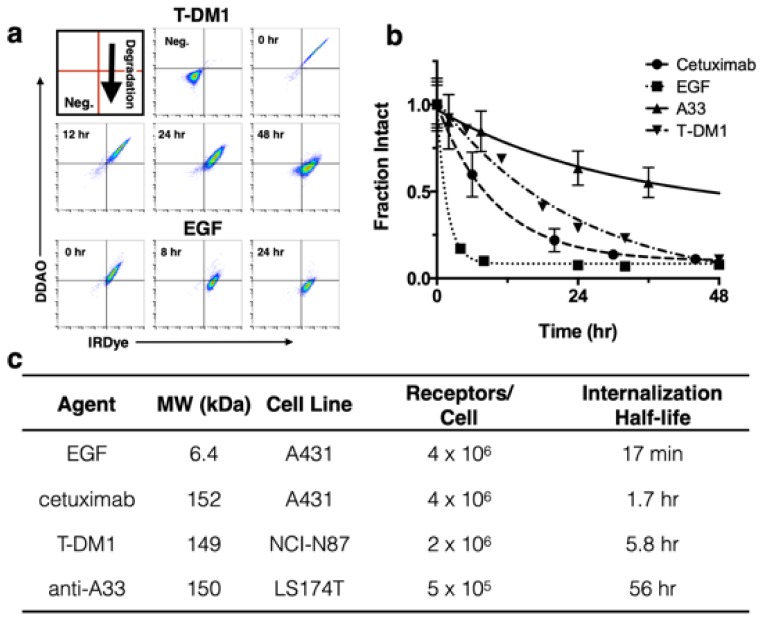
In vitro flow cellular degradation. (**a**) Representative Log-Log flow cytometry plots of dually labeled T-DM1 and EGF gated on cells. Intact protein appears in the DDAO(+)/IRDye(+) quadrant. Over time as the biologic is degraded, there is a gradual shift towards DDAO(-)/IRDye(+). (**b**) Fraction of intact protein for four agents over time. EGF shows rapid internalization and degradation, while A33 maintains signal over several days. Cetuximab and T-DM1 decrease at a moderate rate as expected. (**c**) Model system for validation of dual channel technique. For each model protein the molecular weight and plasma clearance is listed. The associated cell line used for xenografts, receptor density, and internalization half-life (as reported in the literature [24,25,26]) are also listed. Reproduced with permission from reference [8].

For dual labeling, the method requires two NIR fluorescent dyes that have substantially differing residualization rates [15] and do not overlap spectrally (Figure 1). In this work, we discuss two spectrally compatible fluorescent dye pairs IRDye800CW/DDAO and AF647/BoDIPY-FL. (The non-residualizing dye Atto-740 did not have adequate stability in vivo in our hands for reliable measurements.) After the labeled protein binds to a surface receptor, it is internalized, and subsequently degraded. Low molecular weight degradation products labeled with DDAO/BoDIPY-FL and IRDye/AF647 are released. The low molecular weight, lipophilicity, and moderate pKa allow non-residualizing dyes (DDAO/BoDipy-FL) to passively diffuse out of the cell upon protein degradation, while the larger, highly charged, hydrophilic residualizing dyes (IRDye/AF647) remain trapped in lysosomes (Figure 1B). DDAO/BoDIPY-FL, therefore, approximates the intact protein, since it is cleared upon degradation, while IRDye/AF647 approximates the cumulative uptake in the cell, since it is ‘trapped’ within the cell. This method was chosen over alternative mechanisms, such as pH effects [22] or quenching/FRET, because it is irreversible (unlike pH effects) and does not require a high degree of labeling or larger dye-quencher conjugate. Antibodies can be labeled with IRDye800CW/AF647 while having a negligible impact on clearance over the first few days [23], and the additional DDAO/BoDIPY-FL label does not change the clearance (Figure 1C). Previously, the dual technique was applied to the ADC T-DM1 to quantify the number of antibodies internalized per cell [8]; to further demonstrate the utility of this technique we applied it to three other model compounds with widely differing internalization rates (Figure 2). EGF is rapidly internalized and degraded with a 17 min half-life [24], cetuximab and T-DM1 are slower at approximately 2 and 6 h half-lives, respectively [24,25], and the tight-junction associated A33 target and antibody is the slowest with a 56 h half-life [26]. (Note that EGF and cetuximab both target the same receptor, highlighting how the protein and receptor both influence the overall internalization rate.)

To demonstrate application of the method with several biologics in vitro, T-DM1 [8], cetuximab, and A33 were dually labeled with DDAO and IRDye, while a 1:1 ratio of EGF-DDAO to EGF-IRDye was used. Figure 2A shows example flow cytometry plots at various times for T-DM1 and EGF following a 30 min cell labeling. As protein is degraded, the DDAO/IRDye ratio decreases as the intact signal approaches zero. In Figure 2A the intact protein appears in the DDAO(+)/IRDye(+) quadrant and, as it is gradually degraded, shifts to DDAO(-)/IRDye(+). To measure the intact fraction, the cells were analyzed on flow cytometry and the median fluorescence intensity for each channel (DDAO and IRDye) was measured at different times (Figure 2B). Then each channel was normalized to the initial time point, and the fraction intact was calculated from the DDAO to IRDye ratio. This value yields the approximate ratio of intact protein to cumulative uptake. EGF showed rapid internalization and degradation, cetuximab and T-DM1 were degraded at a moderate rate, and A33 maintained strong signal over several days. These degradation rates agreed well with the internalization half-lives of these proteins (Figure 2C) [24,25,26].

**Figure 3:**
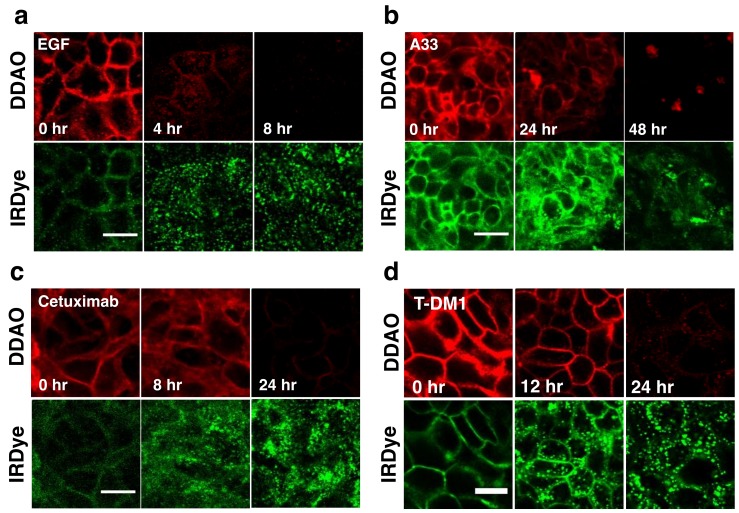
In vitro confocal microscopy of dually labeled (**a**) EGF, (**b**) A33, (**c**) Cetuximab, and (**d**) T-DM1. DDAO (red) shows cell surface labeling with a loss of signal over time. IRDye (green) shows initial cell surface labeling followed by the formation of punctate spots as it is trapped in the lysosomes. Scale bar is 10 µm.

To visualize the dual label technique in vitro, cells were imaged using a confocal microscope at similar time points. Figure 3 shows separate and merged channels for DDAO (red) and IRDye (green) for EGF, A33, Cetuximab, and T-DM1. All four agents showed similar behavior but differed in the time scale for degradation (which matched flow cytometry, Figure 2). IRDye signal is initially at the surface but as the protein is internalized and degraded, it becomes trapped in endosomes and lysosomes, resulting in the formation of punctate spots [15]. Similarly, DDAO labels the surface initially; however, as it is degraded, the dye leaks out of the cells as seen by a drop in signal. Although DDAO does lose some fluorescence due to pH effects (pKa = 5) [28], lysed cells show very low levels of DDAO within the cell lysate (Figure S1) indicating that the loss of the DDAO dye from the cell dominates over pH effects [15]. The ability to detect protein degradation with the BoDIPY-FL/AF647 dye pair was also tested with several antibodies. Cetuximab, T-DM1, and Trastuzumab all displayed a rapid drop in non-residualizing fluorophore (Bodipy-FL, red) and endosome/lysosome trapped residualizing fluorophore (AF647, green) for these systems (Figure S2).

**Figure 4:**
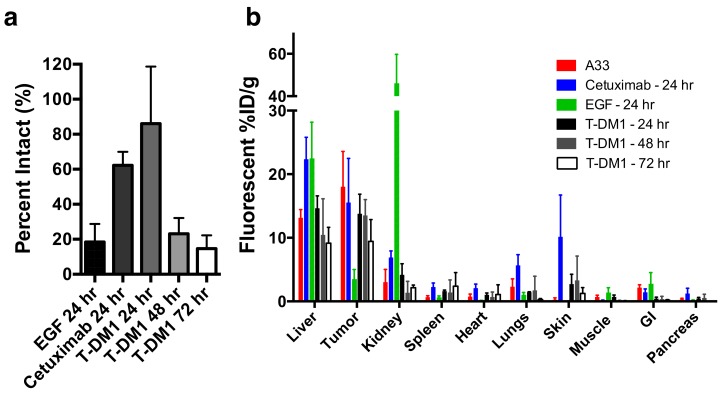
In vivo cellular degradation and distribution. (**a**) T-DM1, EGF, and cetuximab degradation in tumor cells. At 24 h EGF (a rapidly clearing protein), is mostly degraded in the tumor. However, the slowly clearing cetuximab and T-DM1 show mostly intact protein. Over 48–72 h, after maximum uptake is reached, T-DM1 is increasingly degraded. Data plotted as mean ± standard deviation. (**b**) Fluorescence biodistribution of EGF, cetuximab, and A33 at 24 h and T-DM1 at 24, 48, and 72 h.

Applying the dual NIR labeling technique (DDAO/IRDye) to EGF, T-DM1, and cetuximab in vivo yielded insight into the single-cell and tissue distribution of these proteins in vivo (Figure 4). For example, the clinical dose of T-DM1 (3.6 mg/kg) does not fully penetrate the tumor tissue [8,23,29] and only targets approximately 10% of cells by flow cytometry [8]. This penetration depth did not change after 7 days [23] indicating that the therapeutic drug likely never reaches all the tumor cells. However, T-DM1 is clinically approved and effective in breast cancer, indicating that despite this heterogeneity, it still shows a clinical response.

**Figure 5:**
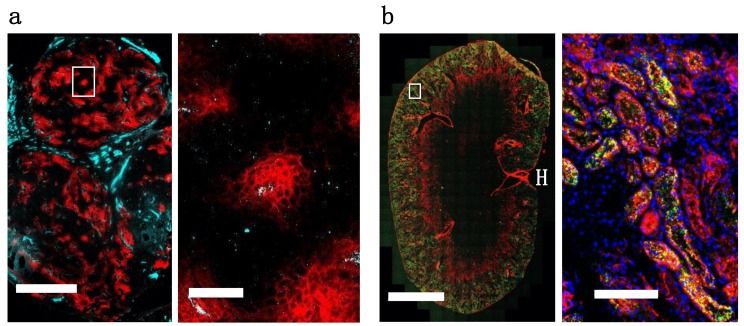
Whole organ immunofluorescence histology. (**a**) High resolution images of A431 xenograft frozen sections 24 h post-injection of 100 µg (~4 mg/kg) of dual labeled cetuximab (DDAO, red). Sections were stained with anti-CD31-AF488 (cyan) ex vivo. Left scale bar is 1 mm and right is 100 µm. (**b**) High resolution images of kidney frozen sections 24 h post-injection of EGF-DDAO (red) and EGF-IRDye (green). High magnification sections were stained with Hoechst 33342 (blue) ex vivo. Left scale bar is 2 mm and right is 100 µm. H = hilum of kidney.

Examining the DDAO/IRDye ratio in vivo through flow cytometry shows how the systemic delivery of the protein plays an important role in the intact versus aggregate degraded probe. EGF, which is cleared rapidly from the blood (Figure S3) and internalized rapidly in vitro, shows little intact protein at 24 h post-injection (Figure 4A). However, the slowly clearing antibodies cetuximab and T-DM1 show mostly intact protein (a ratio of ~1) at 24 h post-injection (Figure 4A) from having a constant intact supply from the blood and an initial time to accumulate in the tumor, even though in vitro the fraction intact decreased significantly after 24 h (Figure 2B). Only at 3 days, once the plasma concentration is lower and after maximum tumor uptake (Figure S3), is the majority of the ADC degraded (Figure 4B) corresponding to maximum payload release. Understanding these kinetics is crucial for many ADCs since they only release their toxic payload after degradation, and cell trafficking is a potential mechanism of resistance [30]. When administering a similar dose of dually labeled cetuximab (100 µg, ~4 mg/kg), fluorescence microscopy shows the tumor distribution is heterogeneous and perivascular in A431 tumor xenografts (Figure 5A). As expected, the small protein EGF, which is below the renal filtration cutoff and accumulates in the kidneys (Figure 4B and Figure S4), localized in the renal cortex (Figure 5B). For smaller therapeutic proteins, the dual label technique can track the distribution in the kidneys, an important clearance organ.

## 4. Discussion

The complex pharmacokinetics and pharmacodynamics of biologics requires a detailed understanding of the distribution in animal models to better translate results to the clinic. Currently, there is a wide array of tools used to study protein distribution at multiple length scales [4]. For example, basic measurements of plasma clearance can be determined using radiolabels, fluorescent labels, ELISA, and mass spectrometry to give blood concentration over time. Using radioactive labels and/or nuclear imaging, the organ uptake and whole animal distribution can be measured in the form of percent injected dose per gram measurements as well as real time imaging with PET/SPECT. Histology can be performed using autoradiography, immunohistochemistry, and immunofluorescence to understand the tissue scale distribution. Flow cytometry and in vitro measurements are used to quantify fluorescence at the single cell scale. However, these methods have several limitations. Biochemical and biophysical techniques, such as ELISA and mass spectrometry, have limited spatial resolution (ELISA) and/or difficulty in measuring low concentration proteins in complex samples (e.g., mass spectrometry imaging). Radiolabels cannot achieve cellular and subcellular resolution and are more cumbersome to use than fluorescent labels due to radioactive half-life time constraints and safety/licensing issues. Immunohistochemistry has a limited ability to measure cellular kinetics, and most immunofluorescence techniques only measure intact protein, meaning the cumulative degradation of a therapeutic cannot be quantified. The residualization properties of other visible light dyes, such as FITC, have been used to study protein degradation [10]; however, the greater autofluorescence in this region of the visible spectrum can reduce sensitivity for organ biodistribution and in vivo imaging. None of these methods are capable of measuring cellular kinetics in vivo, and a combination of different techniques with several animals is needed to obtain data on all the relevant length scales.

A major strength of this dual-labeled fluorescence technique (Figure 1) over conventional nuclear imaging is the ability to readily quantify protein uptake with single cell resolution and absolute quantification (proteins per cell) in vivo using flow cytometry. Although techniques with radiolabeled probes are approaching the single cell level [31], the path length of the positron and/or imaging equipment intrinsically limits the resolution. Also, with therapeutic proteins and antibodies, the in vivo rate of degradation is a critical design characteristic [32]. The rate at which the protein is degraded in vitro and in vivo determines how long the active intact protein can achieve its therapeutic effect on the cell surface and in endosomes [33], or, in the case of ADCs, the small molecule (i.e., payload) release rate.

Applying the dual label technique to model proteins in vitro showed differences in cellular uptake and degradation as quantitated through flow cytometry (Figure 2) and visualized through fluorescence microscopy (Figure 3). By flow cytometry the intact fraction of protein, or degradation rate, was measured and corresponded well with other reported internalization rates of the proteins (Figure 2B,C). Specifically, the rapidly internalized EGF had the fastest degradation rate, followed by cetuximab, T-DM1, and anti-A33. Visualizing the technique in vitro through fluorescence microscopy agreed with these results (Figure 3). Examining the cell lysates by gel electrophoresis confirmed that the proteins were degrading over time and lysine-IRDye adducts were released (Figures S1 and S5).

In vivo, we used the dual label technique to study protein distribution from cellular to organ scale, as well as cellular scale tumor degradation. When T-DM1 was administered at the clinical dose of 3.6 mg/kg we previously found that only around 10% of cells in the tumor were targeted with ADC [8]. Additionally, we found that maximum T-DM1 uptake in the tumor was reached around 24 h post-injection (Figure 4B), indicating most of the tumor did not receive therapeutic. Examining the cells targeted by the ADC showed that most ADC in the tumor was still intact 24 h post-injection. Only after 48 and 72 h was most of the ADC in the tumor degraded (Figure 4A) corresponding to toxic DM1 payload release. Similarly, the slowly clearing cetuximab was mostly intact 24 h post-administration (Figure 4A) and exhibited a highly heterogeneous distribution in A431 tumor xenografts (Figure 5A). EGF, which is cleared more quickly than the antibodies (Figure S3) and is rapidly internalized (Figure 2B), was mostly metabolized in the tumor by 24 h (Figure 4A) and localized to the cortex in the kidney (Figure 4d, Figure 5b and Figure S4). Taken together, these examples show how dually-labeled biologics can provide systemic (plasma) clearance data, organ biodistribution, tissue-level heterogeneity, and single-cell uptake measurements.

The current method has several limitations and potential areas for improvement. Similar to radiolabeling, NIR fluorescence measures the distribution of the dye and not the protein itself [34]. These dyes could be subject to drug transporters following degradation and release, similar to small molecule drugs and ADC payloads. Excessive surface labeling with fluorophores can change the physicochemical properties of the protein, thereby changing the plasma clearance and/or distribution [35,36]. Although significant differences in plasma clearance do not occur at early times for several antibodies that have been tested [37] (Figure S3), following the antibodies over days to weeks can result in faster clearance rates [23]. For longer PK studies, we previously found that the clearance of antibodies singly labeled with AF680 at a dye to protein ratio of 0.3 or less was similar to unlabeled antibody; however, the single label does not have the ability to discriminate intact from degraded protein. Therefore, the dual label technique is better suited for shorter studies. As with negatively charged and/or radiolabeled antibodies [38,39], care must be taken to not over-label the protein. In this study, the degree of labeling was kept very low, around 0.3 for IRDye and 0.7–1.0 for DDAO to minimize the impact of the dyes and better approximate the true protein distribution [19,23]. Basic quality control measures must be performed to ensure the labeling efficiency, lack of free dye, and no loss in binding affinity. Finally, the fluorescence intensity of DDAO was not large enough to measure the bulk organ biodistribution accurately. DDAO was selected based on its rapid washout rate from cells following degradation [15]. However, its optical brightness is lower than the cyanine-based dyes. The IRDye signal is much higher than the background at this wavelength, but for DDAO, the higher 650 nm autofluorescence and lower dye brightness results in low signal. Specifically, the trends behaved as expected in the homogenized organs (e.g., the ratio of DDAO to IRDye was low in the kidney of mice injected with EGF), but the contrast to noise ratio was too low for useful bulk measurements. The DDAO signal is therefore limited to flow cytometry and fluorescence microscopy of cells and tissues.

The use of a residualizing dye is helpful to quantify the total payload uptake when studying the cellular distribution of ADCs. However, it is important to note that the non-site specific lysine conjugation chemistry provides a ‘bulk’ measurement of protein degradation. While this is useful for some applications, such as ADCs with non-cleavable linkers like TDM-1 that require whole antibody degradation, ADCs that utilize cleavable linkers may require specific labeling chemistries to mimic the linker cleavage. Designing surrogate fluorescent linkers can provide a direct measure of linker cleavage in real-time [40].

The organ-level biodistribution using a residualizing NIR fluorescent label matches the values used in radiolabeling [20]. However, the method is more labor-intensive (e.g., organ homogenization) and less sensitive than radiolabeled biodistribution measurements. As mentioned above, the DDAO signal was not sufficient to measure the biodistribution for this non-residualizing label. In addition, the high tissue scattering, even with NIR light, results in low resolution whole animal fluorescence imaging. Therefore, radiolabeling is much better suited for measurements solely focused on organ level distribution and/or whole animal imaging.

The sensitivity of the technique (e.g., lowest number of proteins/cell that can be detected) is highly dependent on the equipment used for the experiment. The specific excitation and emission wavelengths and bandwidths, type of detection, optical path properties, etc. all impact the signal to noise ratio (SNR). However, we have included results on these dye pairs due to the relatively common 635 nm (and 488 nm) lasers used in flow cytometry and confocal microscopy (Figure S6). This often results in 647 nm dyes providing high sensitivity. The autofluorescence and variability of blue-shifted dyes (e.g., green-fluorescence) and the often lower quantum yields on common detectors in the NIR range (e.g., beyond 800 nm) can lower sensitivity above and below the wavelengths used for 647 nm dyes. With the equipment used in this work, the limit of detection For IRDye800CW was ~20,000 antibodies/cell, while AlexaFluor 647 was ~5000 antibodies/cell. With site-specific labeling of high affinity peptides, we have detected down to ~1000 receptors per cell [41]. This level of sensitivity is needed for some targets given the > 100-fold differences in expression in ADC targets, for example [42]. Because of the spectral overlap of DDAO with the higher sensitivity AF647 dye and instability of Atto740, we selected a visible light dye for a non-residualizing partner. BODIPY-Fl was chosen due to the bright fluorescence and lack of pH effects in the endosomal pathway that are prevalent for fluorescein (pKa ~ 6.4). It also has a substantial spectral separation from AlexaFluor 647 compared to red dyes and can readily be excited by common 488 nm laser lines. However, with an appropriate excitation source and controls for overlap, other visible light dyes that demonstrate high membrane permeability, such as TAMRA [27], may be useful for dual labeling.

## 5. Conclusions

The dual label NIR fluorophore technique provides absolute protein uptake and degradation with single cell resolution in vivo using widely available flow cytometry equipment, an achievement currently not possible with other imaging modalities. In addition, it enables multi-scale understanding of distribution in the *same* animal. Antibodies are known to distribute heterogeneously throughout tumors [32,43], and combined with tumor microenvironment heterogeneity, such as differences in vascularization, macrophage infiltration, necrosis, and animal-to-animal variability, this can make comparisons between animals especially difficult. In tumors that have differing vascular density, the overall delivery of the antibody will change and could significantly influence the clinical outcome. Using this technique, in vivo degradation at the cellular scale and distribution at the cellular, tissue, organ, and whole animal scales is done in the same animal. The combination of flow cytometry data for single-cell degradation and uptake with the tissue distribution better informs how novel protein therapeutics acting at the microscopic scale affect the tissue distribution and ultimate response.

## Supplementary Materials

The following are available online at https://www.mdpi.com/1999-4923/12/2/132/s1, Figure S1: NIR Fluorescence SDS-PAGE of dual labeled T-DM1, Figure S2: In vitro confocal microscopy of BoDIPY-FL & AF647, Figure S3: Plasma clearance, Figure S4: Whole animal imaging, Figure S5: NIR Fluorescence SDS-PAGE of dual labeled T-DM1, Figure S6: Sensitivity of fluorescent dyes.
