# Supplementary material for: Practical Guide for Quantification of In Vivo Degradation Rates for Therapeutic Proteins with Single-Cell Resolution Using Fluorescence Ratio Imaging
Source: Pharmaceutics. 2020 Feb 5;12(2):132. doi: 10.3390/pharmaceutics12020132 (PMC7076450; doi:10.3390/pharmaceutics12020132)
Supplement: Supplementary file 1 [file pharmaceutics-12-00132-s001.pdf]

Absolute Quantification of In Vivo Therapeutic Protein Degradation with Single Cell Resolution Using Near-Infrared Ratio Imaging

Ian Nessler[1,2], Cornelius Cilliers[1,2] and Greg M. Thurber[1,3]

[1] Department of Chemical Engineering, University of Michigan, Ann Arbor, MI 48109

[2] These authors contributed equally to the publication

[3] Department of Biomedical Engineering, University of Michigan, Ann Arbor, MI 48109

Supplementary Data

Supplemental Figures – S1 to S6

Supplemental Figures

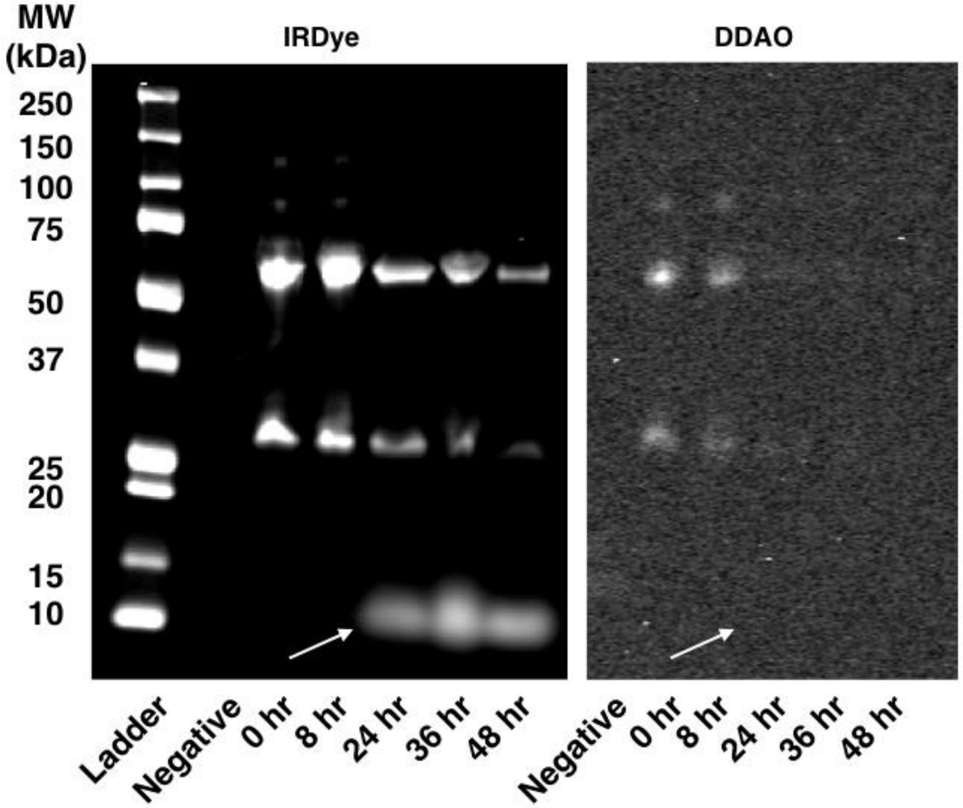

Figure S1 – NIR Fluorescence SDS-PAGE of dual labeled T-DM1. Similar to Fig. 2 and 3, NCI-N87 cells were pulsed for 30 minutes with dual labeled T-DM1 at the corresponding time points. Cell lysate was run on a reducing SDS-PAGE gel and scanned using the NIR Odyssey CLx Scanner. At later times the residualizing IRDye shows formation of lysine-dye adducts (left arrow), while the non-residualizing DDAO does not (right arrow).

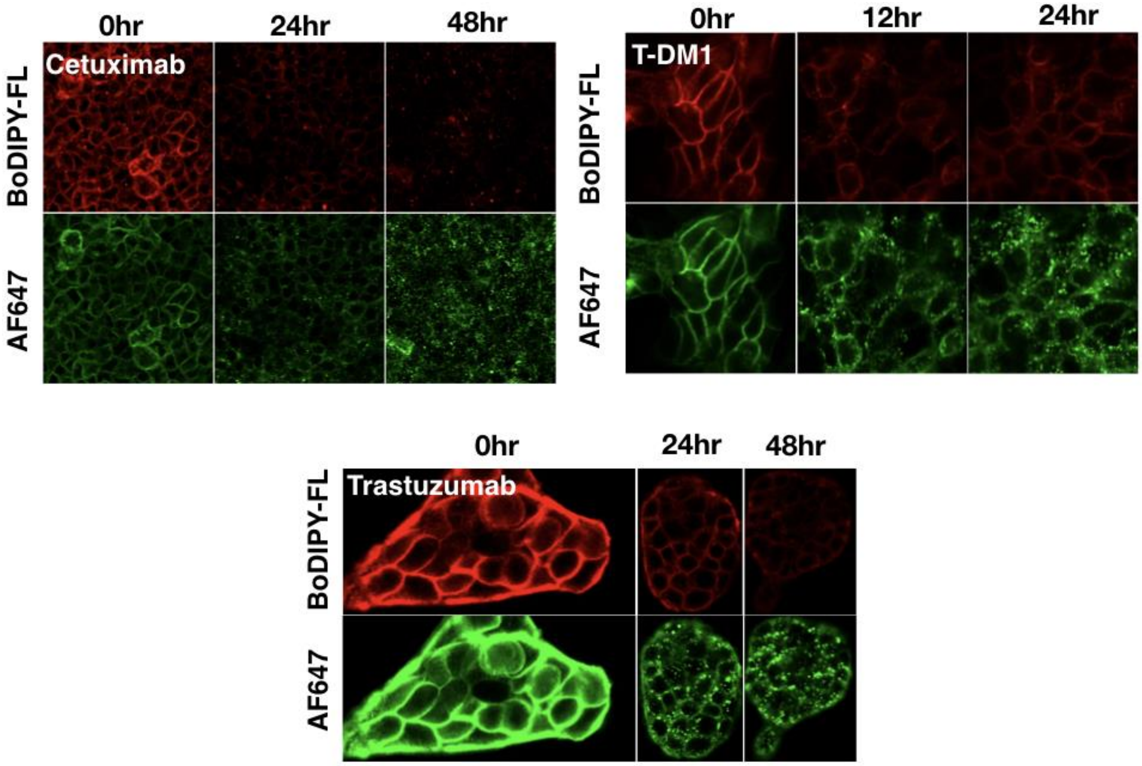

Figure S2 - *In vitro* confocal microscopy of dually labeled Cetuximab (a), T-DM1 (b), and Trastuzumab (c) using AFF647 and BoDIPY-Fl dyes. BoDIPY-FL (*red*) shows cell surface labeling with a loss of signal over time. AF647 (*green*) shows initial cell surface labeling followed by the formation of punctate spots as it is trapped in the lysosomes.

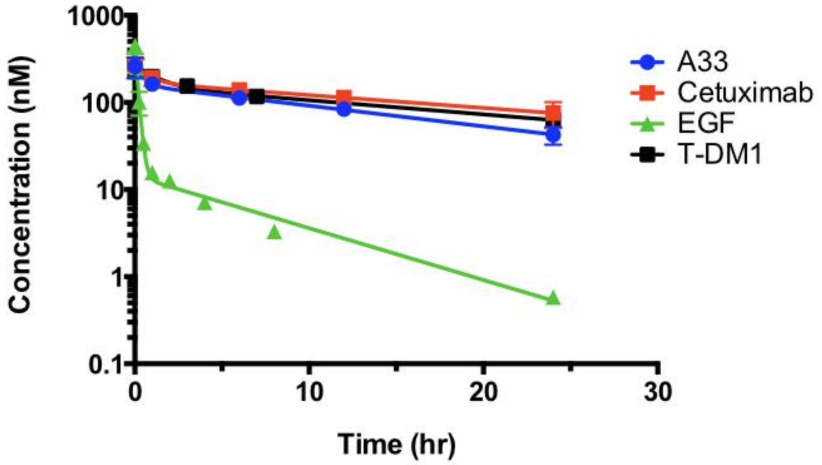

Probe	alpha (hr^{-1})	beta (hr^{-1})	fraction alpha (%)
EGF	6.141	0.14	96.8
cetuximab	1.29	0.03	36.7
A33 MAb	2.08	0.05	39.8
Kadcyla	0.76	0.04	43.1

Figure S3 – Plasma clearance for each agent over 24 hours. Biexponential fits were performed using PRISM and the fitted alpha, beta, and fraction alpha parameters are listed.

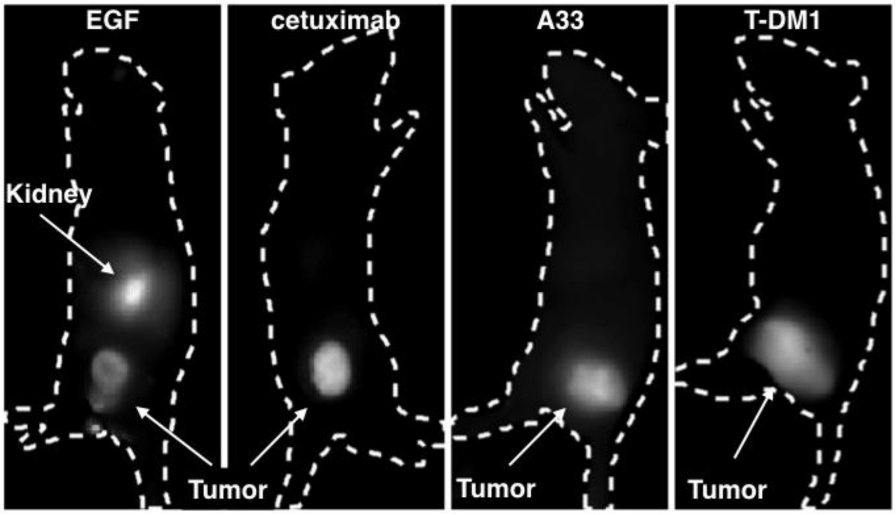

Figure S4 – Whole animal imaging 24 hours post injection for each agent using the PerkinElmer In Vivo Imaging System (IVIS). The low autofluorescence in the near-infrared and residualizing properties of IRDye make it suitable for whole animal imaging. In agreement with the organ biodistribution from Fig. 4b, the three antibodies show high tumor uptake, while the EGF shows high renal uptake and limited tumor uptake.

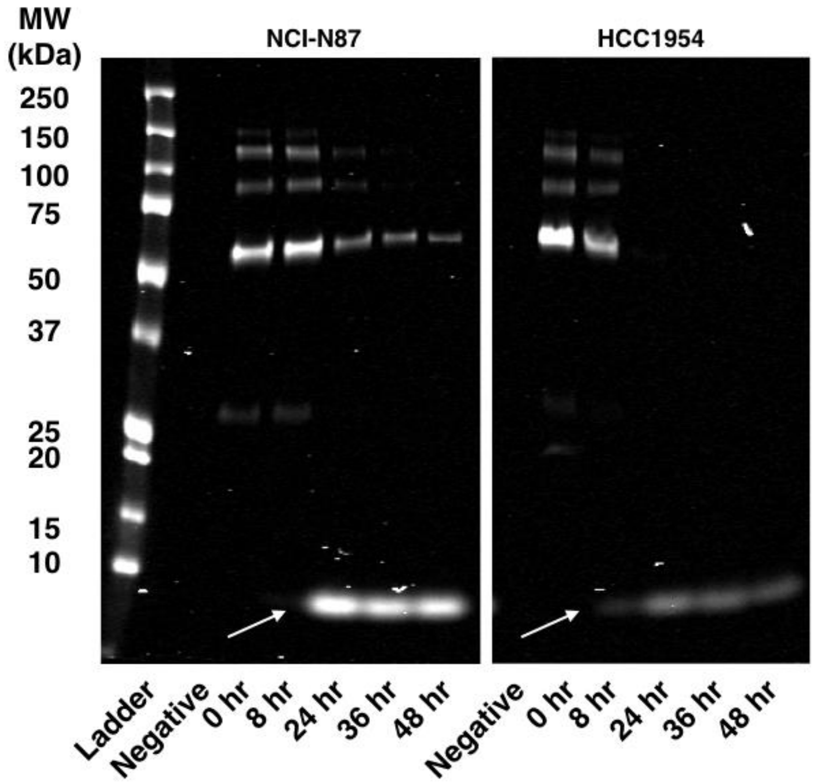

Figure S5 – NIR Fluorescence SDS-PAGE of dual labeled T-DM1. NCI-N87 and HCC1954 cells were labeled for 30 minutes with dual labeled T-DM1 at the corresponding time points. Cell lysate was run on a reducing SDS-PAGE gel and scanned using the NIR Odyssey CLx Scanner. Both cell lines show intact antibody at early times and a gradual formation of lysine-dye adducts at longer times (arrow).

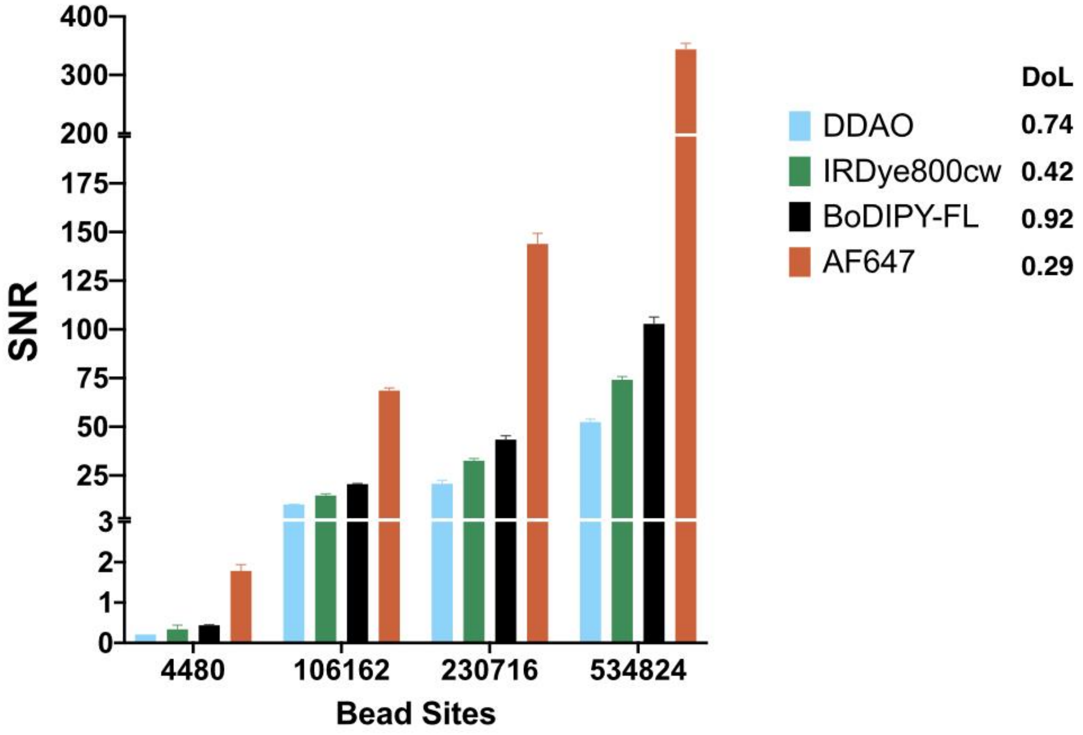

Figure S6 – Sensitivity of fluorescent dyes were compared on flow cytometry. Antibodies were conjugated with fluorescent dyes at the degree of labeling (DoL) shown above and used to label quantitative beads. The SNR is measured by taking the labeled beads (with number of antibody binding sites on the x-axis), subtracting control beads (with no binding sites), and dividing by the background variability *measured on mammalian cells*. The background variability in autofluorescence from cells is the relevant noise when measuring single-cell signals *in vivo*. Because of the better alignment of the 488 nm and 635 nm lasers (along with the detector sensitivity, optical properties of the dyes, etc.), the SNR was highest for AF647, followed by BoDIPY-Fl on this instrument.
